# Isolation and Fractionation of the Tobacco Stalk Lignin for Customized Value-Added Utilization

**DOI:** 10.3389/fbioe.2021.811287

**Published:** 2021-12-06

**Authors:** Zhi Chang Liu, Zi Wei Wang, Song Gao, Yu Xing Tong, Xi Le, Nian Wu Hu, Qun Shan Yan, Xian Gang Zhou, Yan Rong He, Lei Wang

**Affiliations:** ^1^ China Tobacco Hubei Industrial Co., Ltd., Wuhan, China; ^2^ Hubei Xinye Reconstituted Tobacco Development Co., Ltd, Wuhan, China; ^3^ Applied Technology Research of Reconstituted Tobacco Hubei Province Key Laboratory, Wuhan, China; ^4^ Hubei Provincial Key Laboratory of Green Materials for Light Industry, Hubei University of Technology, Wuhan, China

**Keywords:** tobacco stalk, lignin, pretreatment, fractionation, structural interpretation

## Abstract

The value-added utilization of tobacco stalk lignin is the key to the development of tobacco stalk resources. However, the serious heterogeneity is the bottleneck for making full use of tobacco stalk lignin. Based on this, lignin was separated from tobacco stalk through hydrothermal assisted dilute alkali pretreatment. Subsequently, the tobacco stalk alkaline lignin was fractionated into five uniform lignin components by sequential solvent fractionation. Advanced spectral technologies (FT-IR, NMR, and GPC) were used to reveal the effects of hydrothermal assisted dilute alkali pretreatment and solvent fractionation on the structural features of tobacco stalk lignin. The lignin fractions extracted with *n*-butanol and ethanol had low molecular weight and high phenolic hydroxyl content, thus exhibiting superior chemical reactivity and antioxidant capacity. By contrast, the lignin fraction extracted with dioxane had high molecular weight and low reactivity, nevertheless, the high residual carbon rate made it suitable as a precursor for preparing carbon materials. In general, hydrothermal assisted dilute alkali pretreatment was proved to be an efficient method to separate lignin from tobacco stalk, and the application of sequential solvent fractionation to prepare lignin fractions with homogeneous structural features has specific application prospect.

## Introduction

Lignin is generally considered to be a polymer formed by free radical coupling dehydrogenation of three hydroxycinnamyl alcohols (*p*-coumaryl, coniferyl, and sinapyl alcohols), and it is the second most renewable natural terrestrial polymer (Huang et al., 2008; Mahmood et al., 2016). However, lignin is often defined as a by-product of the pulping and biorefinery industries due to its complex structural (Wen et al., 2013b; Han et al., 2021a). The annual production of industrial lignin is up to 60 million tons, but it is often used for low-value thermal energy conversion, and the high value utilization rate is less than 2% (Aro and Fatehi, 2017). In addition, lignin is gradually being regarded as inferior raw material for obtaining heat energy due to its high carbon dioxide emissions during combustion (Ogunkoya et al., 2015). Therefore, the inefficient use of lignin not only wastes valuable resources, but also brings an inevitable burden to the natural environment.

The development of high-value commercial products with lignin as raw materials is the key to the sustainable development of lignocellulosic resources, which has tremendous economic and environmental benefits (Ma et al., 2021a; Han et al., 2021b). Lignin is the unique non-petroleum resource that can provide renewable aromatic compounds, and it has various active functional groups such as hydroxyl, methoxy and carboxyl groups (Lee et al., 2019). Therefore, lignin is considered to be an excellent raw material to replace petroleum-based polymers to commercial products. For example, it has been used in dispersants (Xiao et al., 2021), biofuels (Beauchet et al., 2012), adhesives (Ghaffar and Fan, 2014), flocculants (Wang et al., 2020a), phenolic resins (Lupoi et al., 2015), composite film material (Wang et al., 2021b), carbon fibers (Kadla et al., 2002), polyurethane foams (Cinelli et al., 2013), hydrogels and many other fields (Wang et al., 2020b; Ma et al., 2021b). However, most of the research on the preparation of commercial products using lignin is still in the laboratory stage, and a large-scale industrial breakthrough in lignin-based polymer materials has not yet been achieved. The main reason is the structure complexity and heterogeneity of industrial lignin, which is affected by source, processing, extraction and post-treatments (Gioia et al., 2018). The heterogeneity of lignin makes it difficult to prepare the lignin-based product with stable performance. Therefore, refining industrial lignin into components with homogeneous structure and outstanding functional property is essential for the commercial use of lignin.

In order to obtain more uniform industrial lignin, several fractionation methods have been developed in recent years, including membrane separation, pH acid precipitation, gel permeation chromatography, and organic solvent fractionation (Sadeghifar and Ragauskas, 2020). Among them, the fractionation of lignin by membrane separation method requires a large number of filtration membranes, causing high separation cost (Toledano et al., 2010). The efficiency of lignin fractionation by pH acid precipitation method is quite low. Generally, the yield of lignin components is high at a certain pH value, while the yields of lignin components were lower at other pH values (Gigli and Crestini, 2020). The lignin fractionation by gel permeation chromatography requires complicated equipment and operations (Kirk et al., 1969). Fortunately, sequential solvent fractionation of industrial lignin has received more and more attention due to the advantages of simple operation, low cost, solvent recovery, and low energy consumption (Yuan et al., 2009). The procedure of sequential solvent fractionation is based on the order from low to high solubility of lignin, which mainly depends on the cohesive energy and hydrogen-bonding capacity of the solvent (Schuerch, 1952). It can fractionate industrial lignin into more uniform multiple components according to the structural characteristics and physico-chemical properties, which is an attractive method for the refining of lignin. Moreover, the structure of fractionated lignin is fully interpreted through a variety of advanced characterization techniques to establish a reliable relationship between the lignin structure, lignin reactivity, and specific functionalization requirements, which greatly promotes the diversity and customization of industrial lignin utilization.

Tobacco is widely grown throughout the world, and the output of tobacco stalk left after the leafage production is huge, which is often discarded or used for low-value thermal energy conversion (Meng et al., 2015). The recycling of agricultural waste tobacco stalks has important environmental and economic benefits (Wang et al., 2020c; Jiang et al., 2020). Among them, the high-value utilization of lignin is the top priority. Recently, it was reported that hydrothermal-assisted pretreatment is a good approach to prepare homogeneous hemicelluloses or xylooligosaccharide (XOS) from different lignocellulosic biomass (Ma et al., 2021b). In addition, autohydrolysis can facilitate the subsequent delignification process (Wen et al., 2013d; Chen et al., 2017). Based on these investigations, we firstly separated and obtained lignin from tobacco stem by hydrothermal assisted dilute alkali pretreatment. Subsequently, an economical, easy-to-operate and commercially feasible sequential solvent fractionation process was designed and implemented by using cheap and easy-to-recover organic solvents, such as n-butanol, ethanol, methanol, dioxane. The tobacco stalk lignin was successfully separated into five lignin fractions with lower dispersion and more uniform functional groups. Thereafter, the lignin was comprehensively characterized by a variety of characterization techniques, such as FT-IR, GPC, and 2D-HSQC and ^31^P NMR. Furthermore, the antioxidant activity and thermostability of lignin samples were also studied to provide a theoretical basis for their customized high-value utilization.

## Materials and Methods

### Materials

The tobacco stalk was provided by China Tobacco Hubei Industrial Co., Ltd., Wuhan, China. Before use, tobacco stalk was grinded into fine powders (60–80 mesh) and extracted using the mixture of ethanol/toluene for 12 h in order to remove extractives. The other reagents were purchased from Macklin Biochemical Co., Ltd., China.

### Isolation of Lignin

The lignin was isolated from tobacco stalk by combining hydrothermal pre-treatment with alkali post-treatment. In simple terms, tobacco stalk lignin and distilled water were mixed at a solid-liquid ratio of 10 to 1, and then poured into an autoclave for hydrothermal pre-treatment at 170°C for 30 min in order to remove a portion of hemicellulose and improve the subsequent delignification efficiency. The residue obtained from the hydrothermal process was added to a 2% NaOH alkaline solution at a solid-to-liquid ratio of 1–20 and reacted at 100°C for 3 h. Subsequently, the supernatant containing lignin was obtained through filtration, and added to 6 times the volume of acid water (pH = 2). The solid matter was recovered by filtration and washed three times with acid water. Finally, tobacco stalk lignin was obtained by freeze-drying the solid matter.

### Sequential Solvent Fractionation

**FIGURE 1:**
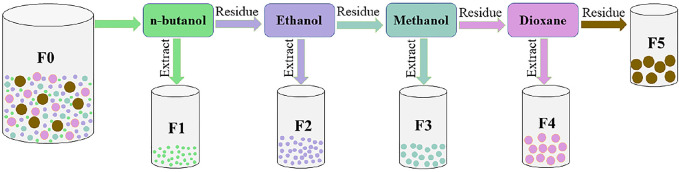
Scheme for sequential organic solvent fractionation of tobacco stalk lignin.

The tobacco stalk lignin was fractionated using organic solvents with lignin dissolution capacity from low to high, which were n-butanol, ethanol, methanol, and dioxane in turn, as shown in Figure 1. First, 50 g tobacco stalk lignin and 500 ml n-butanol were added to a 1,000 ml volumetric flask, and the mixture was stirred at a uniform speed (100 rpm) for 3 h. Subsequently, the solution obtained by filtration was evaporated under reduced pressure and dried to obtain a lignin component soluble in n-butanol, which was noted as F1. The obtained residue was fractionated using ethanol, methanol and dioxane in sequence according to the above steps, and the obtained lignin fractions were F2, F3, and F4, respectively. The resulting insoluble residue was named F5.

### Structural Characterization

The FT-IR spectra, NMR spectra (^2^D-HSQC, and ^31^P NMR spectra), thermogravimetric analysis (TGA) of lignin samples were recorded by Bruker Tensor II spectrometer, Bruker AVIII (400 M), and NETZSCH TG 209 F1 Libra, respectively. The detailed running program and analysis methods of NMR spectra were adopted the procedures and description in the previous literatures (Wang et al., 2017; Ma et al., 2021a). The Mw, Mn, and PDI were measured using the Agilent 1200 GPC system (Ma et al., 2020).

### Thermogravimetric Analysis

The thermal decomposition behavior of lignin was recorded using a thermal analyzer (NETZSCH TG 209 F1 Libra). The tobacco stalk lignin was heated under N_2_ atmosphere with a heating rate of 10°C/min from room temperature to 800°C.

### Antioxidant Activity Analysis

The detailed operation steps refer to previous research (Pan et al., 2006). The radical scavenging activity of 1,1-Diphenyl-2-picrylhydrazyl (DPPH) was recorded using UV-2450 spectrophotometer. Briefly, 0.18 ml of lignin solution (0.01–3 g/L) in 90% aqueous dioxane was added into 0.72 ml of a 25 mg/L DPPH methanol solution at room temperature for 16 min. The concentrations of DPPH radicals at 0 and 16 min were monitored at 515 nm (*λ*max) using UV-2450 spectrophotometer.

## Results and Discussion

### Yields and Molecular Weight Distributions of Lignin Fractions

Regarding the sequential solvent fractionation, the ability of solvent to dissolve lignin is the first consideration when selecting solvents and arranging the extraction order, which is affected by the hydrogen-bonding capacity and the cohesive energy of the solvent. The solubility parameter of solvent can be calculated from the square root of the cohesive energy density, and the shift in wave length can be obtained according to the hydrogen bonding ability (Li et al., 2012). Under the synergistic assistance of solubility parameters with the shift in wave length, coupled with preliminary experimental verification, four organic solvents with increasing ability to dissolve lignin, namely n-butanol, ethanol, methanol, and dioxane, were applied to the sequential solvent fractionation of tobacco stalk lignin.

The yield of the five lignin fractions is shown in Table 1. The yield of lignin fraction dissolved in n-butanol and ethanol (F1 and F2) was 14.59 and 15.4%, respectively. The yield of the lignin fraction dissolved in methanol (F3) was the highest 35.49%, while the yield of the lignin fraction dissolved in dioxane (F4) was 23.18%. The yield of final insoluble residue (F5) was 11.35%. The effect of sequential solvent fractionation on the molecular weight of lignin was explored by GPC technology. The weight-average molecular weight (Mw), number-average molecular weight (Mn) and polydispersity (PDI) of all lignin samples can be seen in Table 1. The weight-average molecular weight of unfractionated tobacco stalk lignin (F0) was 3,155 g/mol, which was much lower than that of natural tobacco stalk lignin. This was because the lignin was degraded and some of the chemical bonds were broken during the hydrothermal-assisted alkaline pretreatment process (Wang et al., 2017a). In addition, the PDI of F0 was as high as 2.96, showing serious heterogeneity, which severely hindered its subsequent high-value applications. After fractionation, the weight-average molecular weights of F1, F2, F3, F4, and F5 were 1,256, 2,988, 4,196, 6,845, and 9,783 g/mol, respectively. It could be found that the molecular weight gradually increased from F1 to F5, which proved that the lignin with small molecular weight had better solubility and was extracted first during the sequential solvent fractionation. The small molecular weight of F1 indicated that it was seriously degraded and the chemical bonds between structural units were destroyed. The excessively high molecular weight of F5 might be due to the severe condensation and the presence of carbohydrates, which would be proved in subsequent analyses (Zhao et al., 2018). In addition, it could be observed that the dispersibility of the fractionated lignin fraction had been greatly improved as compared with F0, which had a positive effect on the high-value utilization (Wang et al., 2021a). In general, the solvent selected and the specified extraction sequence could selectively fractionate tobacco stalk lignin and improve the dispersibility.

**TABLE 1:** Yields and molecular weight distributions of lignin samples.

Lignin sample	Yield (%)	Mn (g/mol)	Mw (g/mol)	PDI
F0	—	1,066	3,155	2.96
F1	14.59	1,005	1,256	1.25
F2	15.40	2,511	2,988	1.19
F3	35.49	3,085	4,196	1.36
F4	23.18	5,611	6,845	1.22
F5	11.35	5,095	9,783	1.92

### Spectral Analysis

**FIGURE 2:**
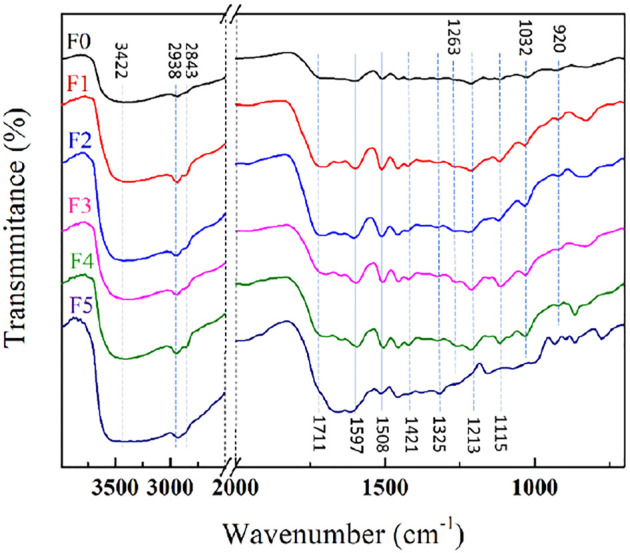
FTIR spectra of tobacco stalk lignin and fractionated lignins.

The alkaline extraction is an efficient method for removing lignin from gramineous plant (Al Arni, 2018). Moreover, the hydronium ion (H_3_O^+^) can be released through the autohydrolysis of water in the hydrothermal pre-treatment process, which could dissolve hemicellulose by breaking the lignin–carbohydrate complexes network and promote follow-up delignification (Garrote et al., 2001; Wen et al., 2013d; Chen et al., 2017). In order to understand the structural evolution of tobacco stalk lignin during the pretreatment process and the effect of the fractionation process on its structure, the FT-IR spectra of the tobacco stalk lignin before and after fractionation were recorded in Figure 2. Previous studies reported that the residue insoluble in dioxane was mainly composed of severely condensed lignin, carbohydrates and ash, and it did not have the potential to produce commercial products (Guo et al., 2013). Except for the final residue insoluble in dioxane (F5), the structure of fractionated lignin was similar to that of unfractionated lignin, but some corresponding signal strengths were slightly different.

The signals at 1,597, 1,508, and 1,421 cm^−1^ are attributable to the aromatic skeleton vibrations, which are signal peaks unique to lignin (Ma et al., 2021c; Xu et al., 2021). This result proved that although the bonds between the structural units would be broken during the hydrothermal assisted dilute alkali pretreatment, the basic skeleton of lignin was not destroyed. The corresponding signals for the vibration of OH groups, the C–H sketch in CH_2_ and CH_3_ groups appeared at 3,422, 2,938 and 2,843 cm^−1^, which were similar in all lignin samples. However, there was a difference in the relative intensities of non-conjugated carbonyl stretching assigned at 1711 cm^−1^. It could be clearly observed that the lignin components with lower molecular weight had a stronger absorption peak at 1711 cm^−1^ as compared to the lignin components with higher molecular weight. This preliminarily proved that lignin components with lower molecular weight had more carboxyl groups, which would be proved in the subsequent characterization. Moreover, the signals at 1,325, 1,213, and 1,115 cm^−1^ belong to S unit, which could be seen in all lignin samples. However, the G unit signals for aromatic ring distributed in at 1,267, 1,029, and 917 cm^−1^ could only be observed in F1-F4, but not in F5. This proved that the lignin component containing more G-type units had better solubility and was fractionated earlier.

**FIGURE 3:**
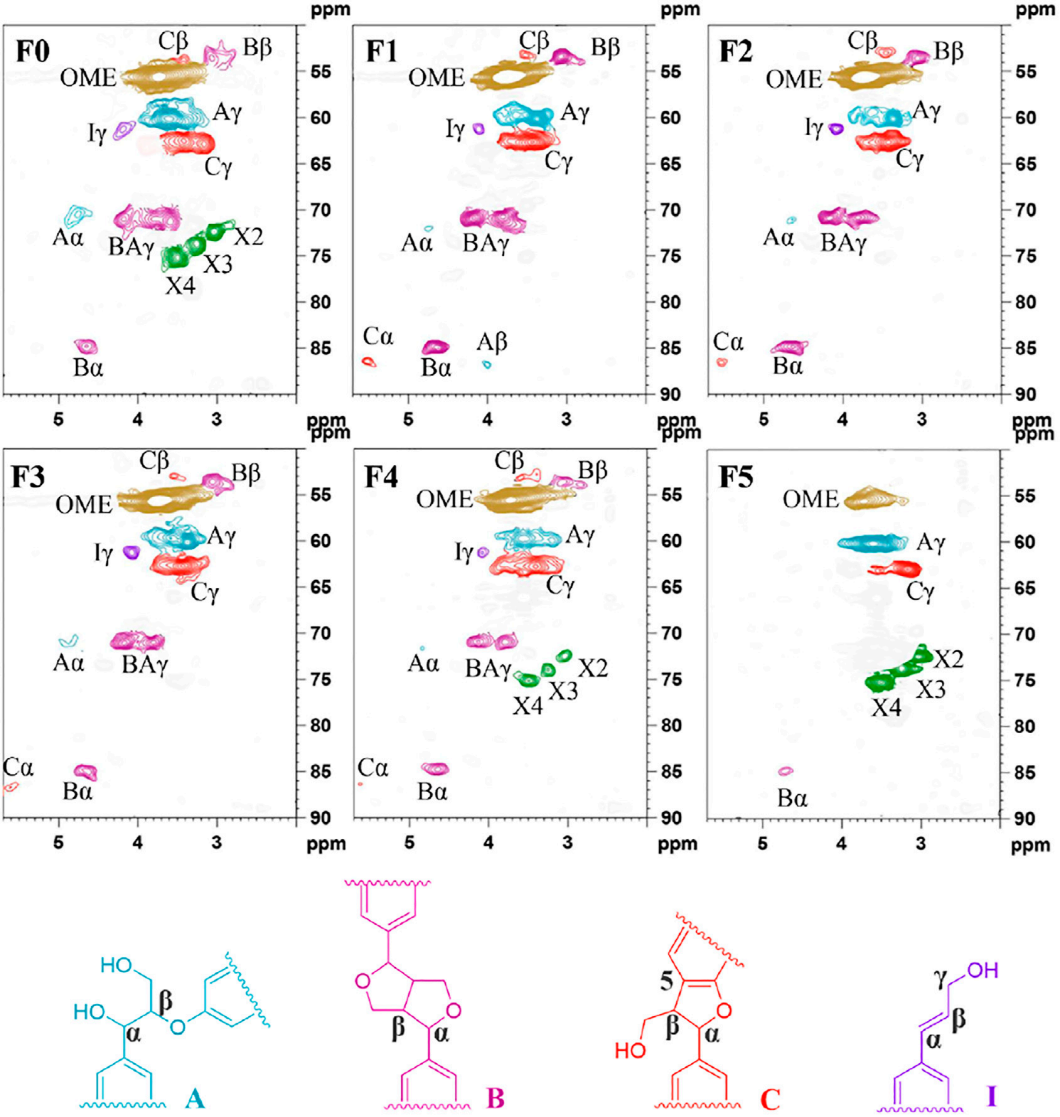
The side-chain of lignin samples in the 2D HSQC NMR spectra.

The comprehensive structural information of lignin can be understood *via* NMR techniques (^31^P and 2D-HSQC), which facilitate the understanding of the structural evolution of lignin in the pretreatment process and the effects of the fractionation process on the structural changes of lignin. The side-chain HSQC spectra (δ_C_/δ_H_ 50–90/2.1–5.7 ppm) of lignin sample was shown in Figure 3. The main cross-signal assignments referred to previous studies regarding to structural characterization of lignin (Wen et al., 2013a; Wang et al., 2017b). In addition to the main C-H correlations of inter-coupling bonds of lignin, such as the methoxyl groups (OCH_3_), *β*-*O*-4 aryl ethers (A), resinols (*β*-*β*, B), and phenylcoumarans (*β*-5, C), and *p*-hydroxycinnamyl alcohol end-groups (I), there were *β*-D-xylopyranosyl signals (X2, X3, and X4) in F0 (Sun et al., 2019). The lignin-carbohydrate complex was formed by combining the lignin at the c-*α*/c-*γ* position and the polysaccharide in substructures *p*-hydroxycinnamyl alcohol end-groups through ether bond. By comparing the spectra of the side chain region of different lignin fractions, it could be found that the carbohydrate signal did not appear in the earlier extracted lignin fractions (F1, F2, and F3), which proved that the purity of these fractions was quite high (Wang et al., 2019). The corresponding signal of carbohydrates could be significantly observed on F5. This proved that F5 contained a lot of carbohydrates, which was one of the reasons for the higher molecular weight.

**FIGURE 4:**
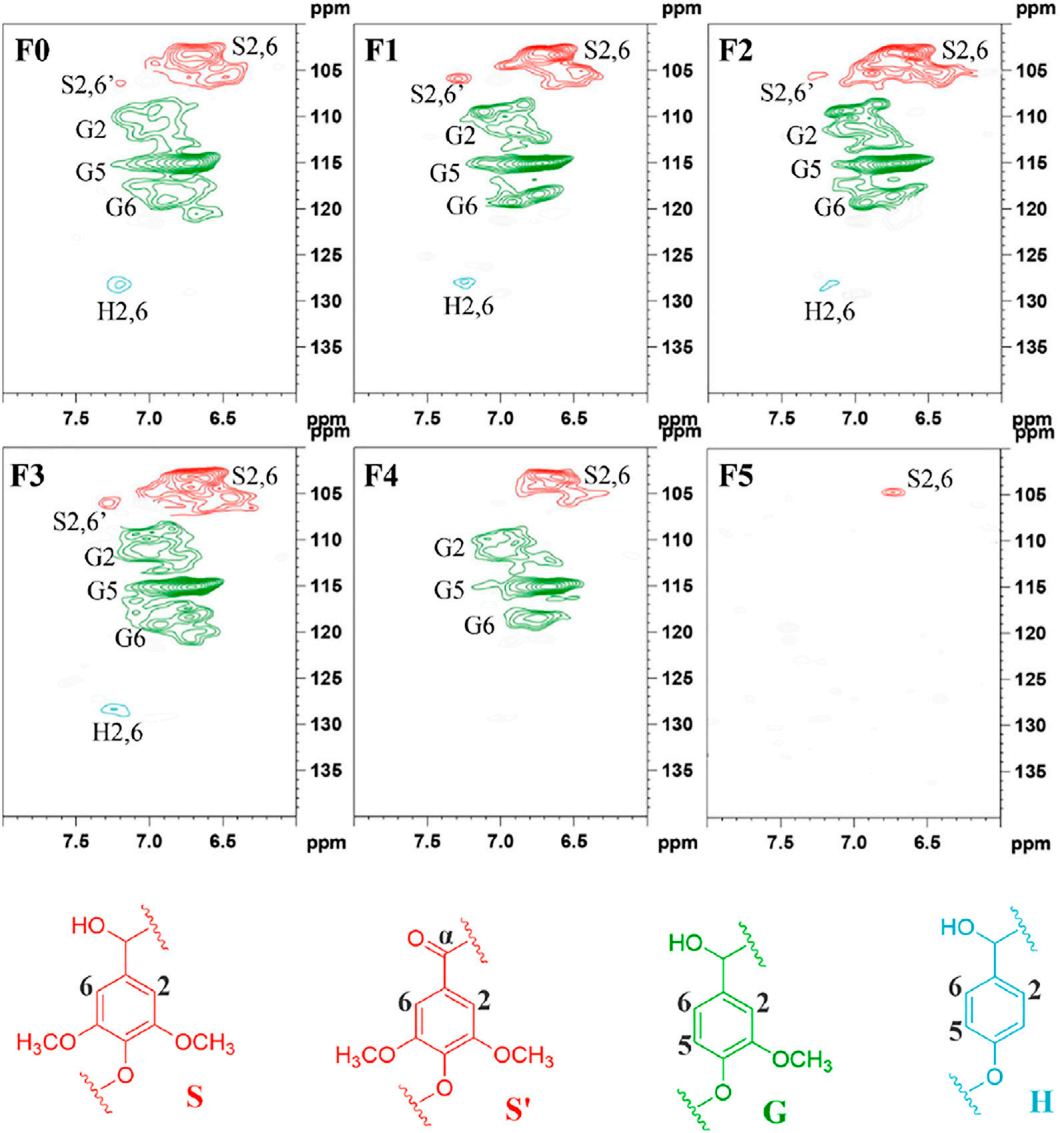
The aromatic ring of lignin samples in the 2D HSQC NMR spectra.

According to the aromatic region (*δ*_*C*_/*δ*_*H*_ 100–140/5.5–8.5 ppm) in Figure 4, cross-signals from guaiacyl (G), syringyl (S), *p*-hydroxyphenyl (H) units were clearly observed (Wen et al., 2013a; Sun et al., 2019), but their signal intensities in each lignin sample were quite different. The normal and C*α*-oxidized S units showed obvious signals for the C_2,6_–H_2,6_ correlations at *δ*_*C*_/*δ*_*H*_ 103.8/6.68 and 86.8/3.96 ppm, respectively. It was worth noting that the trailing of S_2,6_ signal in F0 reflected the condensation of lignin, which was caused by the high temperature during the hydrothermal process. The G units showed three correlations for G6, G5, and G2. Among them, the reason for the double signals on G2 was the serious heterogeneity, which was affected by the substituent group at C4 position of G unit (Shen et al., 2016). Moreover, the structural units of different lignin fractions were different. The lignin fractions (F1, F2, and F3) with more H and G type units had better solubility and could be extracted earlier. Only the weak signal attributed to the S unit could be observed in the F5 spectrum. These results were consistent with the above FT-IR analysis results. In terms of structure, lignin components with more H and G units had better reactivity due to more active sites and smaller steric hindrance (Yang et al., 2015), which was more conducive to the preparation of high value-added products through chemistry reaction.

The quantitative ^31^P NMR spectra of lignin samples were recorded to determine the hydroxyl content of lignin. The corresponding quantitative results were listed in Table 2. Phenolic hydroxyl group is decisive factors for the reactivity and the prospects for commercial application of lignin. Because of the cleavage of *β*-*O*-4 linkages and release of more phenolic OH groups, the tobacco stalk lignin F0 obtained by hydrothermal assisted dilute alkali pretreatment has a high phenolic hydroxyl content (2.41 mmol/g). In addition, it could be found that the phenolic hydroxyl content of lignin was negatively correlated with its molecular weight. The lignin component with a lower molecular weight had more phenolic hydroxyl groups and better reactivity. Among them, the phenolic hydroxyl content of F1 obtained by *n*-butanol fractionation was as high as 3.09 mmol/g. Coupled with the above analysis that F1 has more H and G units, F1 had obvious advantages as a raw material for preparing high value-added products.

**TABLE 2:** Quantification of the lignin samples by quantitative ^31^P-NMR Method (mmol/g).

Lignin sample	AL-OH	S-OH	CG-OH	G-OH	H-OH	Total phenolic−OH	COOH
F0	2.52	0.85	0.19	0.81	0.56	2.41	0.75
F1	1.94	0.92	0.20	1.29	0.68	3.09	1.23
F2	2.59	0.88	0.21	0.98	0.43	2.50	0.85
F3	2.26	0.80	0.15	0.76	0.23	1.94	0.56
F4	2.04	0.69	0.13	0.56	0.24	1.62	0.43
F5	2.68	0.61	0.08	0.38	0.15	1.22	0.15

### Antioxidant Capacity

Lignin is rich in phenolic structure and has antioxidant effect (Dong et al., 2011). To explore the antioxidant capacity, the antioxidant test of lignin samples was performed using DPPH as the radical generator. EC50 is the concentration of lignin when the free radical inhibition rate is 50%. The reciprocal of EC50 can be used to calculate the free radical scavenging index (RSI), indicating that the EC50 and RSI values have a negative correlation. In general, the inhibitory effect of lignin increased with the increase of concentration. The RIS values of F0-F5 were 28.52, 35.63, 30.56, 21.42, 18.37, and 15.22, respectively (Table 3). It was obvious that the antioxidant capacity of lignin was negatively correlated with its molecular weight. Previous studies have demonstrated that the antioxidant capacity of lignin depends mainly on the content of free phenolic hydroxyl groups, followed by other groups such as aliphatic hydroxyl groups and methoxyl groups (Pan et al., 2006; Ma et al., 2021d). Therefore, F1 with the highest phenolic hydroxyl content had the strongest antioxidant capacity, and F1 was an excellent raw material for preparing green antioxidants.

**TABLE 3:** The free radical scavenging index (RSI) of the lignin samples.

Lignin sample	EC50 (μg/ml)	RSI
F0	35.06	28.52
F1	28.07	35.63
F2	32.72	30.56
F3	46.69	21.42
F4	54.44	18.37
F5	65.70	15.22

### Thermal Stability

**FIGURE 5:**
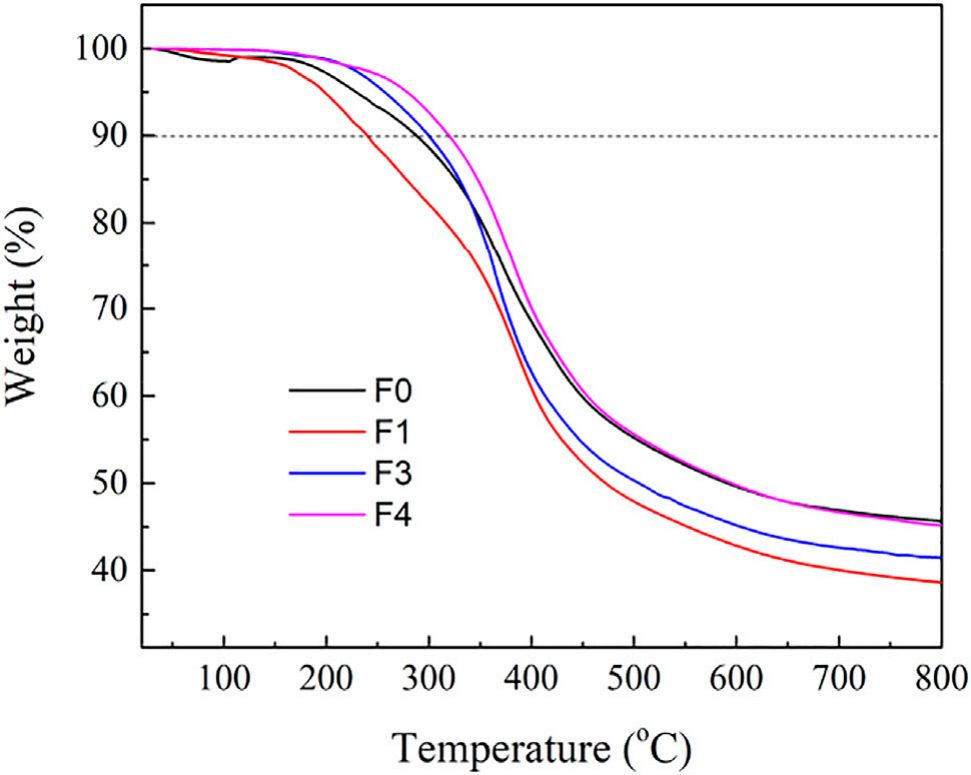
TG curves of tobacco stalk lignin and fractionated lignins.

The thermal behavior of lignin plays a crucial role in the preparation of carbon materials, thermoplastic materials and phenol-rich bio-oils (Sen et al., 2015). In this study, the thermogravimetric analysis (TG) of lignin was used to explore the relationship between thermal stability and structure. The thermal degradation process of lignin is very complex, which is affected by various factors, such as the breakage of internal chemical bonds, the types of functional groups, and the degree of condensation (Xu et al., 2021). Lignin has outstanding thermostability, and its thermal decomposition process can be roughly divided into four stages. In addition, the different thermal stabilities are not only influenced by inherent structure and various functional groups of lignin polymers, degree of branching, and condensation of the lignin macromolecule but also related to specific chemical structures (Wen et al., 2013c). For example, it was reported that the maximum decomposition temperature (TM) was related to the corresponding *β*-*O*-4 ether linkage content and molecular weight (Mw) (Wen et al., 2013c). As shown in Figure 5, the first stage was below 200°C. Dehydration and deformation of weak chemical linkages in the *β*-*O*-4 structure were responsible for the weight loss in this stage (Faravelli et al., 2010). The second stage at 200–350°C was due to the decomposition of ether bonds (mainly *β*-*O*-4 linkages). The subsequent third stage (350–400°C) mainly involved side chain oxidation, such as side chain dehydrogenation, carboxylation of aliphatic hydroxyl group (Ke et al., 2011). The cleavage C-C bond (5-5) and aromatic ring of the lignin caused the fourth stage weight loss at temperatures above 400°C (Yang et al., 2007). Finally, the residue obtained after pyrolysis was mainly composed of inorganic salts and ash.

By comparing the thermal decomposition behavior of each lignin fraction, it could be found that the thermal decomposition behavior of lignin had an excellent correlation with its molecular weight. The decomposition temperatures (10% weight loss) of F1, F3, and F4 were 240, 300, and 320°C respectively, indicating that lignin fraction with low molecular weight was easier to be pyrolyzed. However, the unfractionated F0 (288°C) did not meet this result, which indicated that the thermal decomposition behavior of lignin was not only related to molecular weight, but also affected by many factors, such as heterogeneity, functional groups, and degree of condensation (Gioia et al., 2018). Similarly, the residue weights at 800°C of F1, F3, and F4 were 38.4, 41.4, and 45.9% respectively, which had an excellent positive correlation with molecular weight. However, F0 with lower molecular weight had the highest residual carbon rate of 45.5%. The main reason was the serious heterogeneity of F0 and the existence of ash, making it difficult to use F0 as a promising carbon material precursor. In general, although F4 had inferior reactivity and was difficult to prepare high value-added products through chemical modification, its high carbon residue rate contributed to its application in preparing carbon materials.

## Conclusion

The tobacco stalk lignin was obtained by hydrothermal assisted dilute alkali pretreatment, and the structural features of the lignin were characterized by modern characterization techniques. Subsequently, the tobacco stalk lignin (F0) was divided into five lignin components by sequential solvent fractionation with *n*-butanol (F1), ethanol (F2), methanol (F3), and dioxane (F4). The molecular weight of the lignin elevated with the increase in the solubility of the solvent, and the polydispersity was greatly improved after fractionation process. In addition, the phenolic hydroxyl content in lignin was negatively related to its molecular weight, and the high phenolic hydroxyl content could improve the reactivity and antioxidant capacity. Therefore, lignin components with lower molecular weight (F1 and F2) showed great potentials in the preparation of green antioxidants and high value-added chemical products. By contrast, lignin fraction with high molecular weight was more suitable for preparing carbon materials due to its high residual carbon rate. In short, the full utilization of lignin is the key to tobacco stalk resource recovery. The sequential solvent fractionation can promote the customized utilization of tobacco stalk lignin according to the structural advantage of different lignin fractions.

## Data Availability

The original contributions presented in the study are included in the article/Supplementary Material, further inquiries can be directed to the corresponding authors.

## References

[B1] Al Arni S. (2018). Extraction and Isolation Methods for Lignin Separation from Sugarcane Bagasse: A Review. Ind. Crops Prod. 115, 330–339. 10.1016/j.indcrop.2018.02.012

[B2] Aro T. Fatehi P. (2017). Production and Application of Lignosulfonates and Sulfonated Lignin. ChemSusChem 10, 1861–1877. 10.1002/cssc.201700082 28253428

[B3] Beauchet R. Monteil-Rivera F. Lavoie J. M. (2012). Conversion of Lignin to Aromatic-Based Chemicals (L-Chems) and Biofuels (L-Fuels). Bioresour. Technol. 121, 328–334. 10.1016/j.biortech.2012.06.061 22858503

[B4] Chen T.-Y. Wen J.-L. Wang B. Wang H.-M. Liu C.-F. Sun R.-C. (2017). Assessment of Integrated Process Based on Autohydrolysis and Robust Delignification Process for Enzymatic Saccharification of Bamboo. Bioresour. Technol. 244, 717–725. 10.1016/j.biortech.2017.08.032 28822283

[B5] Cinelli P. Anguillesi I. Lazzeri A. (2013). Green Synthesis of Flexible Polyurethane Foams from Liquefied Lignin. Eur. Polym. J. 49, 1174–1184. 10.1016/j.eurpolymj.2013.04.005

[B6] Dong X. Dong M. Lu Y. Turley A. Jin T. Wu C. (2011). Antimicrobial and Antioxidant Activities of Lignin from Residue of Corn stover to Ethanol Production. Ind. Crops Prod. 34, 1629–1634. 10.1016/j.indcrop.2011.06.002

[B7] Faravelli T. Frassoldati A. Migliavacca G. Ranzi E. (2010). Detailed Kinetic Modeling of the thermal Degradation of Lignins. Biomass and Bioenergy 34, 290–301. 10.1016/j.biombioe.2009.10.018

[B8] Garrote G. Domínguez H. Parajó J. C. (2001). Study on the Deacetylation of Hemicelluloses during the Hydrothermal Processing of Eucalyptus wood. Holz als Roh- und Werkstoff 59, 53–59. 10.1007/s001070050473

[B9] Ghaffar S. H. Fan M. (2014). Lignin in Straw and its Applications as an Adhesive. Int. J. Adhes. Adhesives 48, 92–101. 10.1016/j.ijadhadh.2013.09.001

[B10] Gigli M. Crestini C. (2020). Fractionation of Industrial Lignins: Opportunities and Challenges. Green. Chem. 22, 4722–4746. 10.1039/d0gc01606c

[B11] Gioia C. Lo Re G. Lawoko M. Berglund L. (2018). Tunable Thermosetting Epoxies Based on Fractionated and Well-Characterized Lignins. J. Am. Chem. Soc. 140, 4054–4061. 10.1021/jacs.7b13620 29498848

[B12] Guo G. Li S. Wang L. Ren S. Fang G. (2013). Separation and Characterization of Lignin from Bio-Ethanol Production Residue. Bioresour. Technol. 135, 738–741. 10.1016/j.biortech.2012.10.041 23186676

[B13] Han X. Ding L. Tian Z. Wu W. Jiang S. (2021a). Extraction and Characterization of Novel Ultrastrong and Tough Natural Cellulosic Fiber Bundles from Manau rattan (Calamus Manan). Ind. Crops Prod. 173, 114103. 10.1016/j.indcrop.2021.114103

[B14] Han X. Wu W. Wang J. Tian Z. Jiang S. (2021b). Hydrogen-Bonding-Aided Fabrication of wood Derived Cellulose Scaffold/Aramid Nanofiber into High-Performance Bulk Material. Materials 14, 5444. 10.3390/ma14185444 34576668 PMC8469447

[B15] Huang H.-J. Ramaswamy S. Tschirner U. W. Ramarao B. V. (2008). A Review of Separation Technologies in Current and Future Biorefineries. Separat. Purif. Technol. 62, 1–21. 10.1016/j.seppur.2007.12.011

[B16] Jiang J. Carrillo-Enríquez N. C. Oguzlu H. Han X. Bi R. Song M. (2020). High Production Yield and More Thermally Stable Lignin-Containing Cellulose Nanocrystals Isolated Using a Ternary Acidic Deep Eutectic Solvent. ACS Sustain. Chem. Eng. 8, 7182–7191. 10.1021/acssuschemeng.0c01724

[B17] Kadla J. F. Kubo S. Venditti R. A. Gilbert R. D. Compere A. L. Griffith W. (2002). Lignin-based Carbon Fibers for Composite Fiber Applications. Carbon 40, 2913–2920. 10.1016/s0008-6223(02)00248-8

[B18] Ke J. Singh D. Yang X. Chen S. (2011). Thermal Characterization of Softwood Lignin Modification by Termite Coptotermes Formosanus (Shiraki). Biomass and Bioenergy 35, 3617–3626. 10.1016/j.biombioe.2011.05.010

[B19] Kirk T. K. Brown W. Cowling E. B. (1969). Preparative Fractionation of Lignin by Gel-Permeation Chromatography. Biopolymers 7, 135–153. 10.1002/bip.1969.360070202

[B20] Lee S. Kang M. Bae J.-H. Sohn J.-H. Sung B. H. (2019). Bacterial Valorization of Lignin: Strains, Enzymes, Conversion Pathways, Biosensors, and Perspectives. Front. Bioeng. Biotechnol. 7, 209. 10.3389/fbioe.2019.00209 31552235 PMC6733911

[B21] Li M.-F. Sun S.-N. Xu F. Sun R.-C. (2012). Sequential Solvent Fractionation of Heterogeneous Bamboo Organosolv Lignin for Value-Added Application. Separat. Purif. Technol. 101, 18–25. 10.1016/j.seppur.2012.09.013

[B22] Lupoi J. S. Gjersing E. Davis M. F. (2015). Evaluating Lignocellulosic Biomass, its Derivatives, and Downstream Products with Raman Spectroscopy. Front. Bioeng. Biotechnol. 3, 50. 10.3389/fbioe.2015.00050 25941674 PMC4403602

[B23] Ma C.-Y. Wang H.-M. Wen J.-L. Shi Q. Wang S.-F. Yuan T.-Q. (2020). Structural Elucidation of Lignin Macromolecule from Abaca during Alkaline Hydrogen Peroxide Delignification. Int. J. Biol. Macromolecules 144, 596–602. 10.1016/j.ijbiomac.2019.12.080 31837367

[B24] Ma C.-Y. Gao X. Peng X.-P. Gao Y.-F. Liu J. Wen J.-L. (2021a). Microwave-Assisted Deep Eutectic Solvents (DES) Pretreatment of Control and Transgenic Poplars for Boosting the Lignin Valorization and Cellulose Bioconversion. Ind. Crops Prod. 164, 113415. 10.1016/j.indcrop.2021.113415

[B25] Ma C. Kim T. Liu K. Ma M. Choi S. Si C. (2021b). Multifunctional Lignin-Based Composite Materials for Emerging Applications. Front. Bioeng. Biotechnol. 9, 511. 10.3389/fbioe.2021.708976 PMC8284057 34277593

[B26] Ma C.-Y. Peng X.-P. Sun S. Wen J.-L. Yuan T.-Q. (2021c). Short-Time Deep Eutectic Solvents Pretreatment Enhanced Production of Fermentable Sugars and Tailored Lignin Nanoparticles from Abaca. Int. J. Biol. Macromolecules 192, 417–425. 10.1016/j.ijbiomac.2021.09.140 34582914

[B27] Ma C.-Y. Xu L.-H. Zhang C. Guo K.-N. Yuan T.-Q. Wen J.-L. (2021d). A Synergistic Hydrothermal-Deep Eutectic Solvent (DES) Pretreatment for Rapid Fractionation and Targeted Valorization of Hemicelluloses and Cellulose from poplar wood. Bioresour. Technol. 341, 125828. 10.1016/j.biortech.2021.125828 34461401

[B28] Mahmood N. Yuan Z. Schmidt J. Xu C. (2016). Depolymerization of Lignins and Their Applications for the Preparation of Polyols and Rigid Polyurethane Foams: A Review. Renew. Sustain. Energ. Rev. 60, 317–329. 10.1016/j.rser.2016.01.037

[B29] Meng A. Zhang Y. Zhuo J. Li Q. Qin L. (2015). Investigation on Pyrolysis and Carbonization of Eupatorium Adenophorum Spreng and Tobacco Stem. J. Energ. Inst. 88, 480–489. 10.1016/j.joei.2014.10.003

[B30] Ogunkoya D. Li S. Rojas O. J. Fang T. (2015). Performance, Combustion, and Emissions in a Diesel Engine Operated with Fuel-In-Water Emulsions Based on Lignin. Appl. Energ. 154, 851–861. 10.1016/j.apenergy.2015.05.036

[B31] Pan X. Kadla J. F. Ehara K. Gilkes N. Saddler J. N. (2006). Organosolv Ethanol Lignin from Hybrid poplar as a Radical Scavenger: Relationship between Lignin Structure, Extraction Conditions, and Antioxidant Activity. J. Agric. Food Chem. 54, 5806–5813. 10.1021/jf0605392 16881681

[B32] Sadeghifar H. Ragauskas A. (2020). Perspective on Technical Lignin Fractionation. ACS Sustain. Chem. Eng. 8, 8086–8101. 10.1021/acssuschemeng.0c01348

[B33] Schuerch C. (1952). The Solvent Properties of Liquids and Their Relation to the Solubility, Swelling, Isolation and Fractionation of Lignin. J. Am. Chem. Soc. 74, 5061–5067. 10.1021/ja01140a020

[B34] Sen S. Patil S. Argyropoulos D. S. (2015). Thermal Properties of Lignin in Copolymers, Blends, and Composites: A Review. Green. Chem. 17, 4862–4887. 10.1039/c5gc01066g

[B35] Shen X.-J. Wang B. Pan-Li H. Wen J.-L. Sun R.-C. (2016). Understanding the Structural Changes and Depolymerization of Eucalyptus Lignin under Mild Conditions in Aqueous AlCl3. RSC Adv. 6, 45315–45325. 10.1039/c6ra08945c

[B36] Sun D. Wang B. Wang H.-M. Li M.-F. Shi Q. Zheng L. (2019). Structural Elucidation of Tobacco Stalk Lignin Isolated by Different Integrated Processes. Ind. Crops Prod. 140, 111631. 10.1016/j.indcrop.2019.111631

[B37] Toledano A. García A. Mondragon I. Labidi J. (2010). Lignin Separation and Fractionation by Ultrafiltration. Separat. Purif. Technol. 71, 38–43. 10.1016/j.seppur.2009.10.024

[B39] Wang C. Li H. Li M. Bian J. Sun R. (2017a). Revealing the Structure and Distribution Changes of Eucalyptus Lignin during the Hydrothermal and Alkaline Pretreatments. Sci. Rep. 7, 1–10. 10.1038/s41598-017-00711-w 28377625 PMC5429616

[B40] Wang H.-M. Wang B. Wen J.-L. Yuan T.-Q. Sun R.-C. (2017b). Structural Characteristics of Lignin Macromolecules from Different Eucalyptus Species. ACS Sustain. Chem. Eng. 5, 11618–11627. 10.1021/acssuschemeng.7b02970

[B41] Wang H.-M. Sun Y.-C. Wang B. Sun D. Shi Q. Zheng L. (2019). Insights into the Structural Changes and Potentials of Lignin from Bagasse during the Integrated Delignification Process. ACS Sustain. Chem. Eng. 7, 13886–13897. 10.1021/acssuschemeng.9b02071

[B42] Wang B. Wang H.-M. Sun D. Yuan T.-Q. Song G.-Y. Shi Q. (2020a). Chemosynthesis, Characterization and Application of Lignin-Based Flocculants with Tunable Performance Prepared by Short-Wavelength Ultraviolet Initiation. Ind. Crops Prod. 157, 112897. 10.1016/j.indcrop.2020.112897

[B43] Wang B. Wang S.-F. Lam S. S. Sonne C. Yuan T.-Q. Song G.-Y. (2020b). A Review on Production of Lignin-Based FLocculants: Sustainable Feedstock and Low Carbon Footprint Applications. Renew. Sustain. Energ. Rev. 134, 110384. 10.1016/j.rser.2020.110384

[B44] Wang H.-M. Wang B. Yuan T.-Q. Zheng L. Shi Q. Wang S.-F. (2020c). Tunable, UV-Shielding and Biodegradable Composites Based on Well-Characterized Lignins and Poly(butylene Adipate-Co-Terephthalate). Green. Chem. 22, 8623–8632. 10.1039/d0gc03284k

[B45] Wang B. Sun D. Yuan T.-Q. Song G. Sun R.-C. (2021a). “Recent Advances in Lignin Modification and its Application in Wastewater Treatment,” in Lignin Utilization Strategies: From Processing to Applications. New York: American Chemical Society, 143–173. 10.1021/bk-2021-1377.ch007

[B46] Wang H.-M. Yuan T.-Q. Song G.-Y. Sun R.-C. (2021b). Advanced and Versatile Lignin-Derived Biodegradable Composite Film Materials toward a Sustainable World. Green. Chem. 23, 3790–3817. 10.1039/d1gc00790d

[B47] Wen J.-L. Sun S.-L. Xue B.-L. Sun R.-C. (2013a). Recent Advances in Characterization of Lignin Polymer by Solution-State Nuclear Magnetic Resonance (NMR) Methodology. Materials 6, 359–391. 10.3390/ma6010359 28809313 PMC5452107

[B48] Wen J.-L. Xue B.-L. Xu F. Sun R.-C. Pinkert A. (2013b). Unmasking the Structural Features and Property of Lignin from Bamboo. Ind. Crops Prod. 42, 332–343. 10.1016/j.indcrop.2012.05.041

[B49] Wen J.-L. Sun S.-L. Xue B.-L. Sun R.-C. (2013c). Quantitative Structures and thermal Properties of Birch Lignins after Ionic Liquid Pretreatment. J. Agric. Food Chem. 61, 635–645. 10.1021/jf3051939 23265413

[B50] Wen J.-L. Sun S.-N. Yuan T.-Q. Xu F. Sun R.-C. (2013d). Fractionation of Bamboo Culms by Autohydrolysis, Organosolv Delignification and Extended Delignification: Understanding the Fundamental Chemistry of the Lignin during the Integrated Process. Bioresour. Technol. 150, 278–286. 10.1016/j.biortech.2013.10.015 24184648

[B51] Xiao X. Jiang J. Wang Y. Wang B. Yuan T.-Q. Shi Q. (2021). Microwave-Assisted Sulfonation of Lignin for the Fabrication of a High-Performance Dye Dispersant. ACS Sustain. Chem. Eng. 9, 9053–9061. 10.1021/acssuschemeng.1c02148

[B52] Xu R. Du H. Wang H. Zhang M. Wu M. Liu C. (2021). Valorization of Enzymatic Hydrolysis Residues from Corncob into Lignin-Containing Cellulose Nanofibrils and Lignin Nanoparticles. Front. Bioeng. Biotechnol. 9, 252. 10.3389/fbioe.2021.677963 PMC8085415 33937224

[B53] Yang H. Yan R. Chen H. Lee D. H. Zheng C. (2007). Characteristics of Hemicellulose, Cellulose and Lignin Pyrolysis. Fuel 86, 1781–1788. 10.1016/j.fuel.2006.12.013

[B54] Yang S. Wu J.-Q. Zhang Y. Yuan T.-Q. Sun R.-C. (2015). Preparation of Lignin-Phenol-Formaldehyde Resin Adhesive Based on Active Sites of Technical Lignin. J. Biobased Mat Bioenergy 9, 266–272. 10.1166/jbmb.2015.1514

[B55] Yuan T.-Q. He J. Xu F. Sun R.-C. (2009). Fractionation and Physico-Chemical Analysis of Degraded Lignins from the Black Liquor of Eucalyptus Pellita KP-AQ Pulping. Polym. Degrad. Stab. 94, 1142–1150. 10.1016/j.polymdegradstab.2009.03.019

[B56] Zhao B.-C. Xu J.-D. Chen B.-Y. Cao X.-F. Yuan T.-Q. Wang S.-F. (2018). Selective Precipitation and Characterization of Lignin-Carbohydrate Complexes (LCCs) from Eucalyptus. Planta 247, 1077–1087. 10.1007/s00425-018-2842-9 29350280

